# A posteriori diagnosis of DRESS syndrome induced by diazoxide in a patient with an insulinoma: a case report and review of the literature

**DOI:** 10.3389/fmed.2023.1196041

**Published:** 2023-08-04

**Authors:** Najoua Lassoued, Wafa Alaya, Sondos Arfa, Mouna Korbi, Ines Lassoued, Soumaya Ben Amor, Fatma Zaouali, Zayneb Farhat, Jihen Chelly, Mohamed Habib Sfar

**Affiliations:** ^1^Department of Endocrinology, Taher Sfar University Hospital, Mahdia, Tunisia; ^2^University of Monastir, Monastir, Tunisia; ^3^Department of Dermatology, Fattouma Bourguiba University Hospital, Monastir, Tunisia; ^4^Department of Gastroenterology, Taher Sfar University Hospital, Mahdia, Tunisia; ^5^Department of Family Medicine, Faculty of Medicine of Monastir, Monastir, Tunisia; ^6^Department of Infectious Disease, Taher Sfar University Hospital, Mahdia, Tunisia

**Keywords:** DRESS syndrome, diazoxide, insulinoma, hypoglycemia, severe cutaneous adverse reaction

## Abstract

The Drug Rash with Eosinophilia and Systemic Symptoms (DRESS) syndrome can be potentially life-threatening. The diagnosis is sometimes difficult since the clinical manifestations may be incomplete or non-specific. Insulinoma is a rare functioning neuroendocrine tumor (NET) of the pancreas. Medical therapy may be needed when surgery is contraindicated, delayed or refused. Diazoxide is widely used to control hypoglycemia in patients with insulinoma. We report a clinical case of an insulinoma in a 85-year-old patient treated with diazoxide with a fatal outcome due to a delayed diagnosis of a DRESS syndrome. This is the first case of DRESS syndrome reported after using diazoxide for insulinoma treatment in our knowledge.

## Introduction

The Drug Rash with Eosinophilia and Systemic Symptoms (DRESS) syndrome is recognized now as one of the severe cutaneous adverse reaction (SCAR). It can be potentially life-threatening with with a mortality rate that can reach 10% (1, 2). Most important step in the management of DRESS is early diagnosis and immediate discontinuation of the suspected drug involved (2). The incidence of DRESS syndrome is between 1/1,000 and 1/10,000 drug exposures (1). The diagnosis is sometimes difficult since the clinical manifestations may be incomplete or non-specific (3).

Insulinoma is a rare NET of the pancreas. Its resection is the only curative treatment. Nevertheless, medical therapy may be needed when surgery is contraindicated, delayed or refused. Diazoxide is widely used to control hypoglycemia in patients with insulinoma (4). Hirsutism and rash are common but manageable adverse effects (4). We report a clinical case of an insulinoma treated with diazoxide with a fatal outcome due to a delayed diagnosis of a DRESS syndrome.

## Case presentation

An 85-year-old patient with a history of sleep apnea syndrome was admitted to the Cardiology Department for atrial extrasystoles with a tachycardia at 120 bpm. He was put on beta blockers and amiodarone. During his hospitalization, the patient presented several episodes of severe hypoglycaemia not felt by the patient and requiring continuous infusion of glucose serum.

The patient was therefore transferred to the Endocrinology Department. The diagnosis of insulinoma was retained clinically in front of Whipple's triad and biologically in front of an insulinemia at 65.15 μU/mL with a C-peptide at 11.9 μg/L (VN = 0.8–4.2) concomitant with venous blood sugar at 0.3 g/L.

The thoracic and abdomen–pelvic CT scan showed a welldefined rounded lesion, measuring 22**/**25 mm, at the posterior part of the head of the pancreas. This lesion is spontaneously isodense, enhancing homogeneously and intensely in the arterial phase. This lesion is in close contact with the inferior vena cava which remains permeable. Biliopancreatic endoscopy ultrasound showed a nodular exophytic lesion of the head of the pancreas with no contact with the main pancreatic duct and no mesenteric vascular invasion.

The patient was a candidate for pancreaticoduodenectomy given the size of the tumor and its deep location. But the surgical treatment was refused in the multidisciplinary consultation meeting because of the perioperative anesthetic risk of this surgery, the close contact of the tumor with the inferior vena cava and the patient frailty. The patient was finally prescribed 300 mg per day of diazoxide and hypoglycemic episodes have disappeared since that.

**Figure 1:**
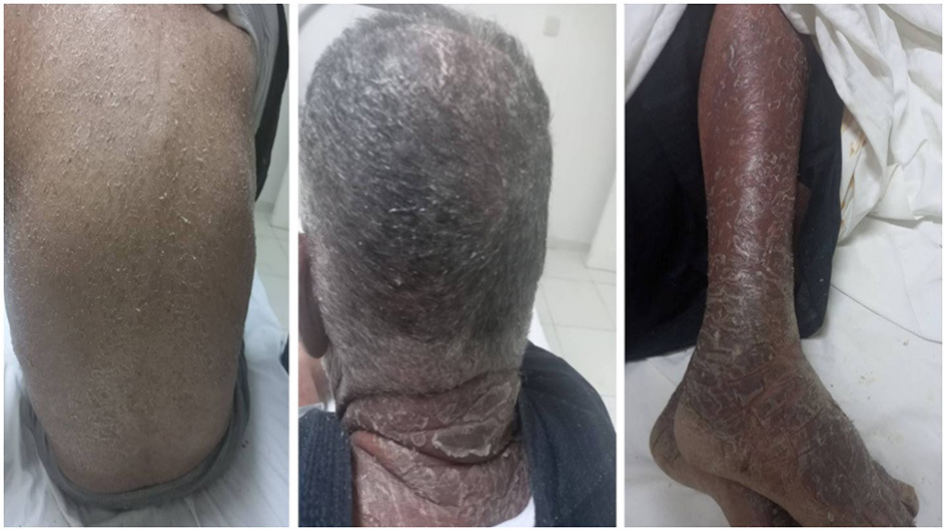
General maculopapular desquamative rash.

One month later, a generalized maculopapular rash developed rapidly progressing to full body associated with exfoliation in thick layers (Figure 1). The patient was not febrile. The rest of physical examination did not reveal any other abnormalities.

The skin biopsy showed the presence of numerous foci of spongiosis with a moderately abundant perivascular inflammatory infiltrate made up of lymphocytes, histiocytes and polymorphonuclear eosinophils. Histopathological examination concluded to drug eruption. On biology, the patient had hypereosinophilia at 1,800/μl, lymphopenia at 1,200/μl and hyperuricaemia at 520 μmol/L. There was no renal failure or hepatic cytolysis.

**Figure 2:**
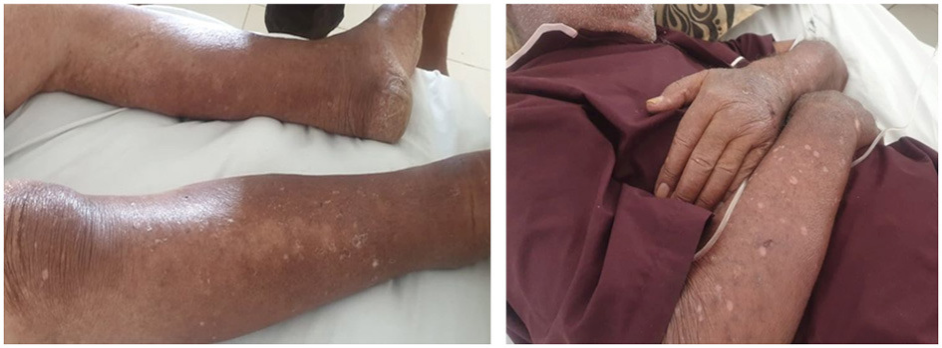
Improvement in skin lesions after 2 weeks of diazoxide withdrawal (upper and lower limbs).

**Figure 3:**
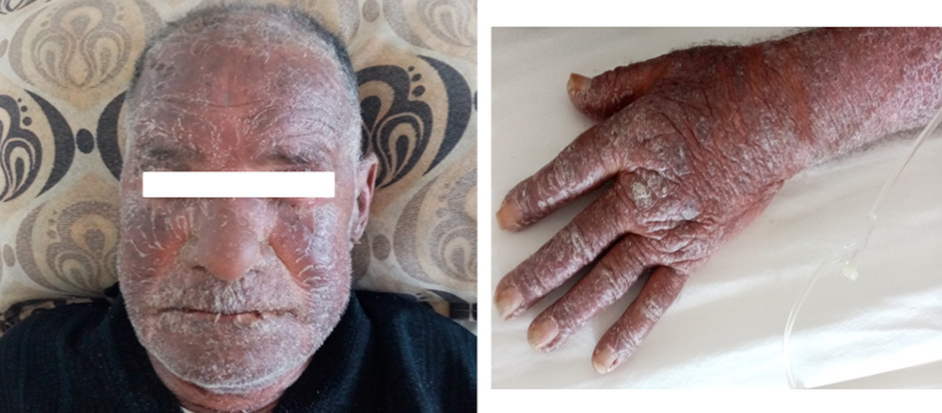
Reappearance of maculopapular desquamative rash.

In view of the persistence of the skin lesions despite bisoprolol and amiodarone withdrawl, we stopped diazoxide and the patient was rehospitalized because of the expected recurrence of hypoglycemic episodes. He was given continuous infusion of glucose 10%. The skin lesions disappeared 52 days after amiodarone discontinuation and 10 days after diazoxide discontinuation (Figure 2). The evolution was marked by recurrence of severe hypoglycemia with recurrent hypokalemia, related to endogenous hyperinsulinism, requiring central venous supplementation. The vital prognosis was at stake because of severe hypoglycemia and hypokalemia. The opinion of the Department of Pharmacology was for the reintroduction of diazoxide with strict monitoring. Faced with the absence of other medical alternatives, in particular the unavailability of somatostatin analogs, we reintroduced therefore diazoxide. Five days later, a generalized scaling maculopapular rash reappeared associated with facial edema (Figure 3). The patient presented with fever, hypotension and acute respiratory distress syndrome. The diagnosis of DRESS syndrome was made and the diazoxide was stopped immediately. Intravenous corticosteroid therapy was prescribed and the patient improved after a few days. The patient died 2 weeks later due to septic shock secondary to catheter-related bloodstream infection (CRBSI).

## Discussion

Insulinoma is a rare NET with an incidence of 4 cases per 1 million persons per year (5). The only curative treatment is surgical excision, according to consensus guidelines of the European Neuroendocrine Tumor Society (6). However, 5–10% of cases are inoperable due to malignancy or tumor recurrence (7, 8). Additionally, some patients are too old and frail at presentation to be suitable candidates for this major surgery (9). In such cases, diazoxide, somatostatin analogs, and the mTOR inhibitor everolimus have been effective in controlling hypoglycemia (6).

Surgery was considered risky in our case. Long-term medical treatment with diazoxide was therefore chosen. Long-term use of diazoxide may be a safe and effective option for the treatment of insulinoma (10). We identified case reports of successful long-term management of insulinoma with diazoxide for 11 years (11) and for 27 years (9). The UK survey of a serie of 40 diazoxide treated insulinomas reported an average duration of treatment of 6 years, with a range up to 22 years (12).

Diazoxide is a non-diuretic benzothiadiazine derivative. It was introduced into clinical practice in the 1960′s as a treatment of hypertension (13). Diazoxide inhibits insulin secretion from pancreatic beta cells by causing the opening of ATP-sensitive K+ channels and by reducing the opening of Ca2+ channels. This leads to a powerful hyperglycemic effect (9).

In 1964, Drash and Wolff (14) reported for the first time the test of the therapeutic effectiveness of diazoxide in severe hypoglycemia. Later on, several authors have reported its efficacy in the treatment of metastatic or inoperable insulinoma. Diazoxide was first approved for use by the United States Federal Drug Agency in 1976 and is approved for the following indications: leucine sensitivity, insular cell hyperplasia, nesidioblastosis, extra malignancy -pancreatic and insular cell adenoma or adenomatosis. Treatment with diazoxide should be initiated at a dose of 150 to 200 mg per day administered in two to three doses; the maximum dose is 600 to 800 mg per day (9).

Side effects are similar to those observed in clinical pharmacology studies with diazoxide in hypertension and reported in the literature on this subject (13). The most serious side effects include fluid retention leading to heart failure, diabetic ketoacidosis and hyperosmolar hyperglycemic state, pulmonary hypertension, thrombocytopenia and neutropenia. Non-serious side effects include hypertrichosis, anorexia, nausea and vomiting, and hyperuricemia (15).

In 1968, Drash noted that side effects of edema and hyperuricemia were most common (60%), hyperglycemia and hypertrichosis occurred in 50%, electrolyte disturbances and ketosis were least common (30 and 20% respectively) (16). In the national UK survey, diazoxide side-effects were recorded in 47% of cases and fluid retention was the most common (30%) (12). Peter et al. reported that the main side effects were, in order of frequency, hirsutism in more than half of cases, ankle edema in half of cases, weight gain in more than a third of cases, and nausea in one tenth of cases (11). In a meta-analysis of articles published from 1947 to 2013 (17), Welters et al. reported data from 644 patients who were treated with diazoxide. Side effects were noted in 50% of patients. Hypertrichosis (52%), fluid retention (30%), vomiting (12%), bone marrow aplasia (3%) and heart failure (3.7%) were the most reported side effects (17).

Thornton et al. reported that the frequency of serious adverse events associated with the use of diazoxide in 165 infants is 9.7% (15).

Our patient presented with DRESS syndrome secondary to diazoxide. He had a RegiSCAR score (18) of 4 (Eosinophilia > 1,500, Rash suggesting DRESS, Rash extent >50% of body surface area, Biopsy suggesting DRESS), i.e., a probable diagnosis of DRESS syndrome. During the reintroduction of diazoxide the evolution was rapid. The diagnosis of DRESS syndrome was made a posteriori with a RegiSCAR score of 6 (Eosinophilia > 1,500, Rash suggesting DRESS, Rash extent >50% of body surface area, Organ involvement) thus a definitive diagnosis of DRESS syndrome.

Set the keyword associated with the terms diazoxide and insulinoma, we performed a literature search of the electronic database MEDLINE from 1969 to 2022 (Table 1). To our knowledge, this is the first reported case of DRESS syndrome after using diazoxide for insulinoma treatment and the first case of DRESS syndrome reported after using diazoxide in general.

**Table 1:** Cutaneous adverse effect reported in adults treated with diazoxide.

**References**	**Number of cases**	**Diazoxide**		**Indication of Diazoxide**	**Cutaneous adverse effect**
		**Duration**	**Dose (mg/d)**		
Warren et al. (10)	1	27 years	400–700	Insulinoma	Hirsutism
Goode et al. (11)	11	5 months	250	Insulinoma	Hirsutism
		3 months	150		
		4 months	200		
		2 years	100		
		3 $\frac{1}{2}$ year	1500		
		3 years	450		
		3 months	300		
		2 years	150		
		8 months	200		
		3 months	300		
		3 weeks	600		Hypersensitivity reaction/ Stevens-Johnson syndrome
Gill et al. (12)	4	6 years (range 1–22)	100–600	Insulinoma	Hirsutism
	1				Rash
Black (13)	4	–	–	Hypoglycemia	Dermatological (maculopapular rash, malar flush, photosensitivity)
	25				Hirsutism
Niitsu et al. (19)	2	42 day	300	Insulinoma	Rash
		15 day	50		
Menter (20)	1	3 months	600	Severe hypertension	Hypertrichosis Lanuginosa and a Lichenoid Eruption

DRESS syndrome is a rare but severe drug rash. Its severity is due to systemic manifestations that can progress to multi-organ failure and be life-threatening. Its physiopathology is now better understood, involving a predisposing immunogenetic background and reactivation of herpesviruses dominated by the HHV-6 virus (21). DRESS syndrome is a delayed drug hypersensitivity reaction, occurring 2–6 weeks after the first intake of the offending drug. The time to onset of this reaction is therefore longer than that observed in most other SCAR (2), and is therefore largely responsible for the frequent diagnostic delay in this syndrome (21).

Many drugs have been implicated, including antiepileptics (phenytoin, carbamazepine, phenobarbital), allopurinol, antiretrovirals (nevirapine), antibiotics (minocycline), and nonsteroidal anti-inflammatory drugs (ibuprofen, phenylbutazone) (1, 2). The survey should look for those whose introduction dates back between 3 weeks and 3 months before the start of DRESS (22). In a systematic review of DRESS cases reported in the literature between 1997 and 2009, a total of 44 drugs were associated with the 172 reported cases (21). In another recent review of the literature published in 2018 (2), diazoxide was never reported to be responsible for DRESS syndrome.

The imputability of diazoxide requires skin tests and a meticulous pharmacovigilance investigation. The skin patch test and lymphocyte transformation test are among the most commonly used investigations (2). Patch testing should be performed 2–6 months after recovery from the symptoms for optimal results (23). The sensitivity and specificity of the lymphocyte transformation test increase if it is performed 5–8 weeks after the onset of symptoms (24). Our patient died before this time.

In any case, these tests often lack specificity (2, 22, 25) hence the notion of chronological imputability which takes into account the evocative delays, the evolution after stopping treatment and the recurrence of drug eruption after reintroduction of the same drug.

## Conclusion

Patients worldwide have been treated with diazoxide for more than 60 years. Hirsutism, hypertrichosis and rash have been considered as the most frequent non severe side effects of diazoxide. This is the first case of DRESS syndrome reported after using diazoxide for insulinoma treatment in our knowledge.

## Data availability statement

The original contributions presented in the study are included in the article/supplementary material, further inquiries can be directed to the corresponding author.

## Ethics statement

Written informed consent was obtained from the individual(s) for the publication of any potentially identifiable images or data included in this article.

## Author contributions

NL: the first named physician of the patient. WA: the second named physician of the patient. SA, MK, IL, SBA, FZ, ZF, and JC: collaboration in patient care. MH: supervising. All authors contributed to the article and approved the submitted version.
